# Geophagia: Benefits and potential toxicity to human—A review

**DOI:** 10.3389/fpubh.2022.893831

**Published:** 2022-07-26

**Authors:** Julius Nsawir Bonglaisin, Noella Bajia Kunsoan, Patrice Bonny, Chelea Matchawe, Bridget Ndakoh Tata, Gerard Nkeunen, Carl Moses Mbofung

**Affiliations:** ^1^Center for Food and Nutrition Research, Institute of Medical Research and Studies of Medicinal Plants (IMPM), Yaoundé, Cameroon; ^2^Centre for Transport and Logistics, “Sapienza” University of Rome, Rome, Italy; ^3^Center for Research in Neurobiology, “Sapienza” University of Rome, Rome, Italy; ^4^Department of Biochemistry, Faculty of Science, University of Dschang, Dschang, Cameroon; ^5^The University of Bamenda, Bamenda, Cameroon

**Keywords:** geophagy, prevalence, toxicity potential, clay, review

## Abstract

Geophagy is the habit of consuming clay soil such as chalk or kaolin. Though it is globally practiced, the safety of those involved is yet to be fully established. It is thought to be highly prevalent in pregnant women because of its antinausea or therapeutic effects. This practice is also thought to be provoked by some nutritional needs, but in modern society its etiology is obscure. The mineralogical and chemical compositions of clay may vary from one region to another and even in all form of rocks clay constitutes. Published articles in geophagy indicate lack of adequate investigations into the toxicity of geophagy, though it is globally practiced and more prevalent in Africa (as a continent) or in Africans migrants. Some studies have helped to identify some minerals that are toxic to human if ingested. In most cases, the potential toxicity emphasized by these studies is based on the detection of the presence of these nuisance elements in the geophagic materials. Scientifically, a lot has been done in the light of detection of toxic matter, but more investigations on metabolic studies are still necessary. The variability of clay content with respect to source motivated this review on geophagy and its potential toxicity to human. This review is aimed at bringing out findings that would enable a better understanding of the toxicity potential of geophagy across context and taxa.

## Introduction

Geophagy or geophagia is the habit of consuming clay such as chalk or kaolin. Commonly referred to as Calabar chalk, ndom, nzu or Calabar stones by some ethnic groups in Nigeria or as mabele by the Lingala people of Congo or by Francophones as craie, poto or argile (1, 2) or Calabar chalk in Cameroon, kaolin consumption varies in intensity from one region to the other. It is more common in children than in adults (3, 4); in women than in men (5, 6); in black race than in white race (5, 7); in rural areas than in urban areas (5, 8, 9); and in pregnant women than normal women (7, 10–12).

In earth science, kaolin is used broadly to cover a range of clay-compounds predominately made up of Kaolinite that is associated with many other minerals that are the products of felspathic rock alteration (13, 14). The Kaolinite and minerals of kaolin vary from sample to sample. Its color varies from white, green, pink, gray, yellow to red with respect to chemical composition. Kaolin is clay material, made of hydrated silicates and known to be stable within natural conditions and its chemical composition, at pure state, is as on Table 1.

**Table 1:** Chemical composition of pure kaolin.

**Constituent/ignition LOI**	**Percentage**
Aluminum oxide (Al_2_O_2_)	37–39
Silica oxide (SiO_2_)	45–47
Ferric oxide (Fe_2_O_3_)	0.4–0.5
Titanium oxide (TiO_2_)	1.9–2.1
Calcium oxide (CaO)	0.4–0.7
Sodium oxide (Na_2_O)	0.6–0.7
Potash (K_2_O)	0.02–0.1
Loss in Ignition LOI	12–14

*By Gamiz et al. (15)*.

It may be contaminated with toxic metals such as lead (Pb), nickel (Ni), cobalt (Co), Cadmium (Cd), chromium (Cr), copper (Cu), Mercury (Hg) Mercury (Hg), zinc (Zn), etc. (13, 16–19).

Kaolin is one of the most abundant clay compound that is also extremely exploited in the world industrially (14). It is highly mined in several countries such as Brazil, Germany, India, China, Bulgaria, Korea, Czech Republic, Australia, South Africa, France, United States of America, Iran and United Kingdom. Kaolin is common in tropical soils especially in soils that are products of chemical weathering of rocks in hot and typically humid climate such as tropical rainforest areas (14).

In Cameroon, kaolin deposits were discovered by Njonfang (20) and Njoya (21) in the following regions: Centre (Etoa, Mvan, Nanga Eboko, etc.); Littoral (Dibamba, Makepe, Mbanga, Dizangué; Douala sedimentary basin, etc.); North West (Bambili, Bali, Mankon, Santa, etc.); South west (Mamfe, Mukunda, Ediki, etc.); West (Mayouom, Balengou, Bana, Lembo, etc.) and in the Northern area of the country (Moufou, Dekounou, Mbe and Gamboukou, Zilling, Doubled, etc.) (20, 21).

Kaolin can be used as filler in the manufacture or production of several goods according to its intrinsic chemical characteristic and the extent to which it is processed. The highest use of this clay is for paper production, especially in ensuring the gloss in paper grades. Kaolin is also used to make toothpaste, ceramics, cosmetics, white bulbs, paints and to reinforce properties of rubber and in adhesives (22, 23). As well, it is used in smoking pipes production in Asia and Europe, in organic farming and applied as spray to crops to prevent damage from insects or sun scald. It is also used like whitewash in traditional stone masonry homes in Nepal, in radiological dating since it contains traces of uranium and thorium and for making of fiber glass, refractories, plastic, mineral wool, etc. (14); for facial masks or soap and to soothe stomach gastritis (24) or for treatment of diarrhea. It is also used for the production of baked bricks and pottery artifacts like in the Bamenda area of Cameroon. The sources and number of published data related to geophagy used in this review are presented on Table 2.

**Table 2:** Source and number of published data related to geophagy.

**Data source**	**Number of studies involved**
Australia	02
Cameroon	08
China	01
England	05
Ghana	06
India	04
Italy	01
Iraq	02
Jamaica	01
Kenya	04
Mexico	02
Nigeria	07
South Africa	11
Tanzania	01
Turkey	03
Uganda	01
USA	08
Vienna	02
Not specified	09
	Total = 78

*Reviews on geophagy: 07*.

## Prevalence, etiology and management of geophagy

Kaolin-eating is common in the rural South of the United States, parts of Latin America, Asia and the Middle East (9, 25, 26). It is also common in Sub-Saharan Africa where several cultures especially farmers and nomad settings consume dirt, mainly clay (5, 27, 28) and in Australia during food scarcity (29). In most cases the habit is common amongst pregnant women. Many experts have suggested that geophagy is highly prevalent in pregnant women, who are either Africans or migrants from this continent, because of its antinausea effects (30–32). Other studies have confirmed this finding through more investigation in women (33, 34). The Medical University of Vienna (35) found that 30–80% of Africans, especially women, eat clayey soil on a regular basis consuming between 100 and 400 g per day. Specifically, 30–50% of women in pregnancy (in hundreds of millions) in many African and rural blacks of South American communities practice clay-eating (33, 34, 36).

In modern societies, geophagy represents eating habit with hidden reasons. Some statements have been raised as reasons for geophagical behavior. Some of these include: (1) clay includes additional micro/macro nutrients that may be absent in the consumer's day to day diet. With this reason, clay would be beneficial to the body if consumed. Such is the case with pregnant women who require 20% more nutrients (37); (2) kaolin or clay has the capacity to detoxify secondary compounds that are often found in foods. Thus clay would be like medicine alleviating some pathologies (38); (3) kaolin or clay prevents the stomach and intestines from harmful biochemical substances and thus ensures resistance to gut disease. Clay is said to have covering role in gastro-enterology (39). These statements are confirmed by studies in some communities, indicating that geophagy could be associated with some positive biochemical effects. Danford (40) tried to describe summarily the four hypotheses most commonly discussed as reasons for clay (kaolin) or soil consumption. These include: nutritional, psychological, cultural and medical or therapeutic. For instance, diarrhea and intestinal parasites have been observed to be treated by ingesting clay or soils (36). Dietary treatment that has been observed to stop the habit of geophagy strongly support the viewpoint point that the practice is prompted by nutritional deficiency (41). However, most of these reports are from specific contexts where studies were carried out and as such cannot be generalized. Thus a generic or global approach without comprehensive data from the various contexts will not be adequate. It would be more appropriate to recommend contexts with comprehensive data, identified as posing no threats for geophagic material. This view point is approved by authors (42, 43) with the standpoint that geophagic material should be accepted as such based on its provenance. Some soils have been identified as posing no threats with respect to heavy metal loads (44).

**Figure 1:**
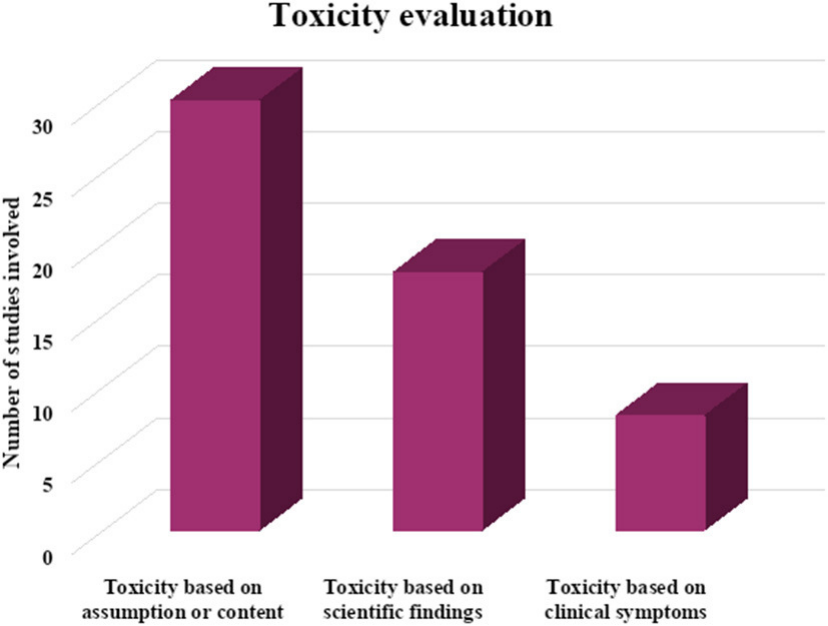
The bases of toxicity of geophagic material in reviewed papers.

Globally, authors have tended to conclude on toxicity based on the content of geophagic materials, such as the presence of heavy metals, instead carrying further scientific investigation for adequate scientific evidence as seen on Figure 1. This is a gap that needs to be addressed for proper apprehension of toxicity in future research on geophagy.

Concerning its management or treatment, Jackson et al. (45) suggest that emphasis should be made on both physiological and psychological aspects of the individual. If geophagy emanates from a physiological health condition or mineral deficiency, a thorough examination of the individual's medical history will reveal potential issues and deficiencies that will be tackled in the treatment process. On the psychological aspect, they recommend an examination of the individual's social history for psychological diagnosis and treatment which is only secondary to the physiological treatment process but essential be considered. Waswa and Imungi (27, 46) carried out a randomized control trial amongst boarding secondary school students in Western Kenya. They used traditional food substitute as a soil replacer within the student community. These were alternatives that help impede the practice of geophagy. Therefore, developing a soil replacer within geophagic communities can be a good management for geophagy. In most cases, geophagy goes unnoticed by various health providers because those who practiced it are reluctant to give information on the habit. Indeed, many have been stigmatized and they tend to conceal their eating habit, making management difficult (47).

### Geophagy and minerals absorption

Though some elements in kaolin within certain thresholds can benefit the body nutritionally for functioning and development (48), some are not essential and might be harmful or toxic instead. The latter are causes of most health challenges encountered during geophagy (49, 50). Those that are important might still be chelated and unavailable nutritionally. Minnich et al. (51) have come up with the hypothesis that kaolin consumption may keep pregnant women from getting enough calories or the right nutrients during pregnancy. The hypothesis is confirmed for some minerals by many scientists. For instance, the role kaolin (clay) plays in iron absorption and assimilation is significantly displayed in studies carried out by some authors (52, 53). They worked with four different types of clay and discovered that Fe ions permeate exchangeably with Ca, Mg, Mn, Na, K and hydrogen ions, leading to the formation of non-absorbable iron compounds. This means that kaolin may sap the body of iron and create anemia. In addition, Dreyer et al. (6) discovered that iron was liberated at pH 2.0 (stomach pH) and absorbed at pH 6.2 (intestinal pH). Hooda et al. (54) used *in vitro* geophagia simulations in five large samples of soils eaten by the communities of Tanzania, Uganda, India and Turkey (54). Both readily available macro (Mg and Ca) and micro (Cu, Fe, Zn and Mn) nutrients necessary for uptake by the gut and the wellbeing of the body constituted these simulation solutions. They found out that Mg, Mn and Ca found in the simulations were released from the soils with no specific order or logic and other simulations with similar soils were not releasing their Mg, Mn and Ca, but instead absorbed them. The absorption of these metals by the clay involved was observed to increase with increased pH. Similar results were obtained by Alloway (55).

Results from many context and taxa reveal that the habit of geophagy can potentially tamper with iron levels in the body in two ways: (i) the Fe content of the geophagic material might make a significant improvement to Fe intake as such alleviating iron deficiency (48) and (ii) The composition of the geophagic material might hinder through chelation non-haem Fe in the gut by forming complexes that cannot be dissolved, influencing negatively Fe absorption as a whole (56–59) and thus affecting iron metabolism. The former point has made researchers to insinuate that lack of Fe in the body provokes geophagia (60) to meet iron (Fe) requirement.

However, Kambunga et al. (61) in their “review of the nature of some geophagic materials and their potential health effects on pregnant women,” concluded that the negative effects of geophagy in pregnant women far outweigh the health benefits. They also noted that the perceived health effects of geophagy in pregnant women are rather based on mere assumptions than scientific experimental data.

### Geophagy and physiological effects

Physiologically, many nutritionists and researchers have viewed geophagy as amongst practices that help provide physical relief from painfulness or distress. In gastro-enterology, clay (kaolin) is suspected to sap toxins from food or bacteria that are related to stomach upset (33, 34, 38, 41, 62). Some kaolin constituents such as benzoic groups, aluminum oxide and magnesium oxide, have attributed antacid characteristics to kaolin, such that it can mitigate pains resulting from gastritis. Furthermore, an author has also observed that kaolin has “covering properties,” and plays a bandaging role in gastro-enterology (39).

Kaolin has been observed to coat the gastro-intestinal tract, preventing it from biological and chemical attacks. It is also potentially therapeutic to esophagitis, colitis and diarrhea (31, 36, 62).

Studies involving some chimpanzees consuming clay-like soil have been observed prior to or after eating some plants possessing anti-malaria properties. Studies that simulated mastication and digestion observed that clay facilitates the release of active anti-malarial components from the leaves of these plants. These soils are also therapeutic toward diarrhea and have been exploited locally for diarrhea treatment (63). According to Tayie et al. (64) some of the motivating and useful reasons for kaolin consumption include their efficiency to alleviate diarrhea, stomach upset or mitigation of nausea and toxemia of pregnancy.

### Geophagy and infections or clinical effects

Authors have reported risks involved in eating earth contaminated with animal or human wastes or even eggs of parasites like in the case of worms, that can remain inactive in the soil for years or even radioactive matter (27, 46, 65). *Clostridium tetani* cells are common in such soils and can pose a further risk. In this light, there is greater susceptibility in children getting infected by bacteria or worms because of their predisposition to soil-eating (66). Authors have also reported that geophagia can bring about teeth damage, the ingestion of contaminated soil, and blockage of human intestines. Studies in Kenya (4) have confirmed these results, with children being the most exposed to geohelminths and trichuriasis (27, 46). Also, incidences of death have been known to occur after the excessive consumption of some clay often due to intestinal occlusion or perforation (5, 22).

A more common problem reported across continents such as Portugal in South America, Iran in the Middle East, Turkey in Eastern Europe, Egypt in North Africa, known as geophagic disease or syndrome observed over 30 years amongst clay consumers was anemia and zinc deficiencies, delayed growth, enlargement of the spleen and liver and retarded sexual maturity (40, 52, 53). Authors have raised the hypotheses that geophagy causes iron deficiency and is also a consequence of lack of iron in the body system (40, 67). The latter hypothesis has been scientifically confirmed (68, 69).

### Geophagy and heavy metal intoxication

Though apparently there are beneficial effects of kaolin consumption presented by many studies, other studies show that this practice does equally tend to expose consumers to metal contaminants and toxicity (2, 70, 71). Amongst these are heavy metals that are permanent and at the same time most problematic. They distinguished themselves from the other contaminants because of their non-degradability and low migration ability in soils. According to their toxicity they could be divided as follow: *strongly toxic:* mercury (Hg), uranium (U), indium (In), cadmium (Cd), copper (Cu), thallium (Tl), arsenic (As), vanadium(V), zinc (Zn), nickel (Ni), bismuth (Bi); *moderately toxic:* manganese (Mn), chromium (Cr), palladium (Pd), lead (Pb), Osmium (Os), tin (Sn), cobalt (Co), molybdenum (Mo), antimony (Sb); and *slightly toxic:* iron (Fe), germanium (Ge). Some metalloids such as selenium (Se) and antimony (Sb), are also examined as heavy metals. Some of these heavy metals are biologically necessary (Fe, Cu, Zn, Se), while others are not (72), because they have no physiological role. Geochemical studies reveal that some of them exist only as traces (Cd, Tl, Hg etc.), but the others exist in high concentrations such as iron (Fe), manganese (Mn) etc (72).

The scenario of clay contamination by heavy metals has been described by several scientists. Clay (kaolin) often develop negative charges within its layers especially in humid and hot environment and consequently attracts positively charged particles. Contamination is the outcome if these positive charges are toxic to humans, like heavy metals (15, 73). Lead would be a good example of a heavy metal that is attracted by such layers of kaolin, consistently reported by many studies (13, 19). Lead occurs naturally in ground waters, surface waters and soils. Studies have shown lead content of agricultural soils varying from 1 to 135 mg/kg and a median value of 11 mg/kg reported (74). Being a water soluble element, it is obvious lead would be leached or washed by rain water into kaolin mines especially those mines that are along or within streams and rivers. A similar scenario would occur when cultivated farm fields are on a slope above kaolin mining sites. In such farms bioavailability and bioaccessibility of heavy metals to crops should be recommended (44, 75). Contamination with lead would be exceedingly high if the stream or river gaining access to the kaolin mines has initially traversed a city or industry (76, 77). Studies have revealed high Pb concentrations in excess of 1,000 mg/kg in soils within most major cities like Lagoes, Douala etc (78–82). Chaney et al. (83) have reported Pb values as high as 50,000 mg/kg within a city. Mostly, these elevated Pb values found in city soils are suspected to come from industrial wastes, vehicles and the various Pb paints (84). The urban environment has thus become a potentially harmful lead (Pb) source. Lead is among the striking environmental harmful chemicals and is becoming preponderant (85). Other sources of lead (Pb) include Pb arsenate found in insecticide, impurities in different forms of fertilizers and metal extracting activities (86).

Previously, research findings on the practice of geophagia in Africa showed mostly the prevalence of the practice and the reasons for it with little or no information on the toxicological implications. Secondly, there has been lack of consensus globally in published literature on the effects of consumption of kaolin on blood heavy metals levels. While studies by Simpson et al. (7) and Thihalolipavan et al. (87) reported that geophagy leads to high Pb blood levels in female consumers, other studies by Knezevich and Tadic (88), Hassen et al. (89), and by Katsumata et al. (90) portray a contrary view. They reported that kaolin consumption tends to be a detoxifier of heavy metals and poisons. Authors confirmed clay ability to adsorb toxins especially when associated with charcoals (91). This apparent lack of consensus on the effect of consumption of contaminated kaolin compels further need for research. This inconsistency inspired Bonglaisin and collaborators to investigate further on the most contaminant heavy metals (Pb, Cd and Hg). In 2011 and 2015, research activities carried out by these authors showed that Clay (kaolin) at the local sale points contained high values of lead (Pb), cadmium (Cd) and Mercury, and is also highly consumed in Cameroon (13, 19). Further research carried by one of the authors on pregnant rats showed that Pb in this clay can gain access in the bloodstream and as such clay (kaolin) is potentially harmful. Cadmium (Cd) and Hg were observed to bio-accumulate in the liver (92). Similarly, serum levels of Pb and varying concentrations of other heavy metals have been found (93, 94), confirming that toxic heavy metals can be absorbed during toxic clay consumption. Ekosse et al. (43) inferred limited health threats in a study in which they observed low bioavailability of heavy metals in gastrointestinal tract simulation. By implication toxicity effects due to Pb would be common in geophagic communities where these studies were carried out. Lead (Pb) is known to be toxic to some organs like the kidney, liver, nerves, gastrointestinal tract, thyroid gland, brain etc. of human or animal systems that ingested it (95–98). Many studies on Pb contamination of kaolin have been reported in Nigeria (1), Ghana (49, 50), South Africa (43), though Pb content or heavy metal potential toxicity of several clay types have not been investigated in other context and taxa (99).

### Geophagy and effects on iron or iodine as micronutrients of public health importance—Example of lead (Pb) contaminated clay

Iron (Fe) and Pb attached themselves on similar position in many biomolecules and so will compete for the same target or binding sites in the human body (100). Though Pb primarily manifests its toxicity in the human body because of its ability to mimic elemental calcium (Ca), it is also known to tamper with Fe absorption and assimilation in mechanism that are yet to be fully known (100, 101). The dislocation of iron (Fe) by zinc (Zn) in oxygen carrying molecule of the human body (hemoglobin), to form Zn protoporphyrin (100), is an initial effect common with Pb intoxication (101). The consequence is reduced oxygen circulation since elemental Fe bears the burden of carrying in the oxyhaemoglobin molecule (102), with outcome being hypochromic anemia. Lead (Pb) is also known to affect negatively erythropoiesis blocking the maturation of red blood cells (100, 101), influencing their size (microcytic anemia) (103) and their longevity (103). In anemia situation, it would block the hormonal response that targets the normal increase of erythopoiesis (100). Studies on geophagic materials have confirmed these findings (68, 69), indicating that toxic clay has the potential to affect hemoglobin level.

Iodine and lead (Pb) manifest a similar scenario. There is a hormonal mechanism controlling the release of thyroid hormones from the thyroid gland. Thyrotropin that is released from the hypothalamus acts on the pituitary gland to release thyroid-stimulating hormone (TSH). The latter (TSH) as its name implies stimulate the thyroid gland to produce thyroxine (T_4_). This hormone (T_4_) regulates TSH output through negative feedback mechanism. When the blood level of T_4_ drops by its conversion to triiodothyronine (T_3_) favored by the enzymes deiodinases (104), the release of pituitary TSH is triggered, with outcome being increased thyroid volume (thyroid hyperplasia). Increased T4 brings about the inhibition of TSH and thyrotropin production. The situation that occurs when sufficient iodide is available in the thyroid gland (102). In iodine deficiency, the level of T_4_ remains low while that of TSH remains high (105).

**Figure 2:**
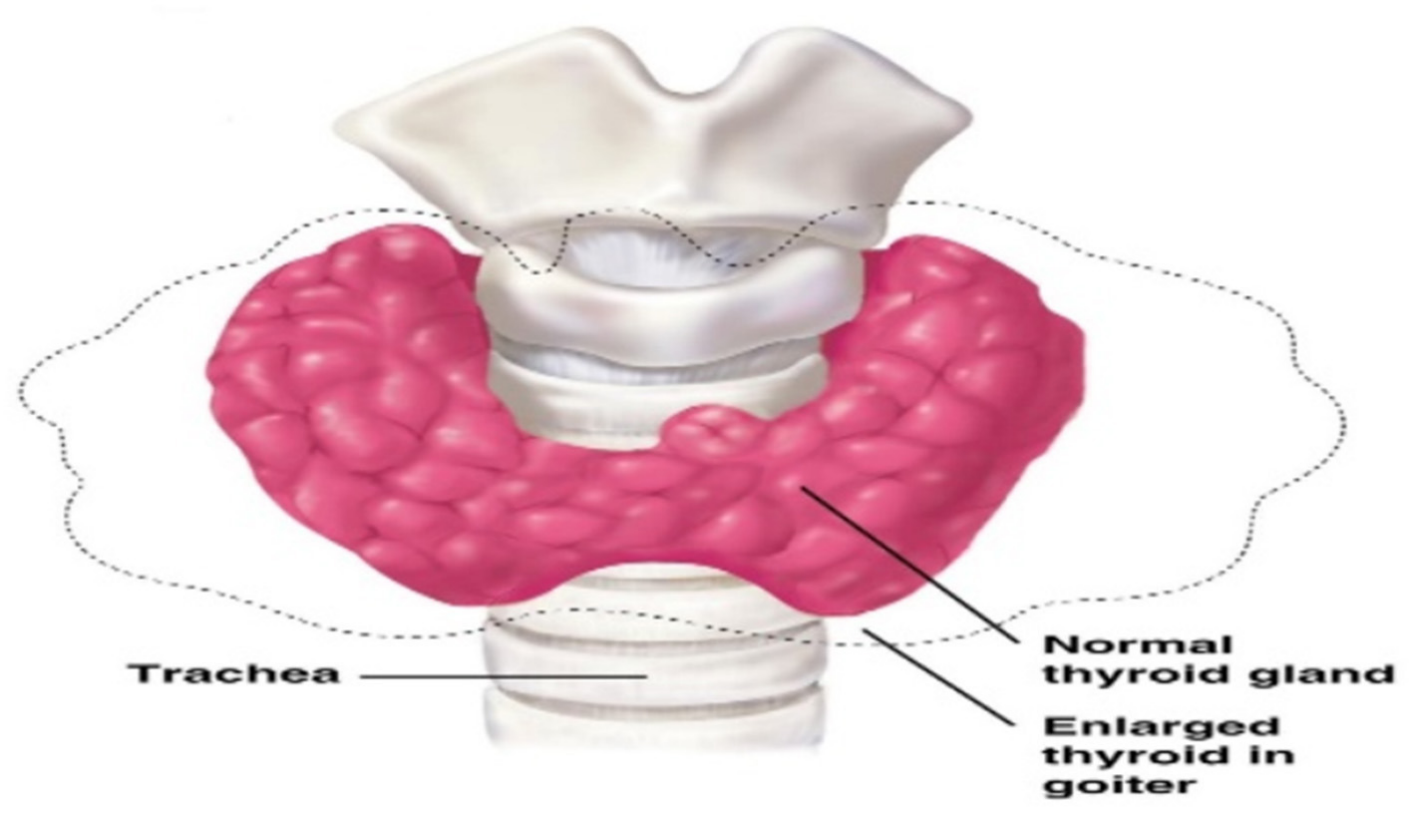
Goiter illustration by the Centre for Disease Control (CDC), USA.

Environmental chemicals have been observed to alter thyroid hormone levels through mechanisms that are well understood such as interruption of iodine transport, disruption of deiodinases etc. (106). Although authors have primarily focused on chemicals that are structurally similar to T_4_ like bisphenol, appreciable attention has also been given to heavy metals such as Pb, Hg and Cd. An association between these toxicants with respect to the total and free T_4_, total and free T_3_, or TSH have been studied (107–112), asserting the negative effects of these metals on the thyroid. Depending on the mechanism that is being disrupted its proper functioning is often affected causing it to hypertrophy even at normal iodine intake as illustrated on Figure 2.

Lead (Pb) for example is known to have negative effects on blood formation (113, 114). Studies on communities with high lead (Pb) exposure have observed its adverse effects associated with free T_4_, or T_3_ (112, 115–117), though there have been inconsistencies as some studies on human revealed no adverse effects on thyroid hormones (118–121).

In sheep, a low dose of lead (Pb) exposure for a long time has confirms Pb effects on thyroid function especially in hypothyroidism (122). A similar study reports increase risk of thyroid damage in wistars albino rats exposed to Pb and Cd (123), with thyroid hypertrophy observed (69) as seen in Figure 2.

### Geophagy and probable lead (Pb) toxicity mechanism—Example of Pb contaminated clay

Though the crystalline structure of kaolin is held together by hydrogen bonding and Wan der waals forces (124), lead (Pb) type in kaolin determines its bioavailability in the human system. In kaolin all evidence points on ionic form that is held by electrostatics forces (as previously mentioned), and readily available for absorption (15, 73). Generally, the main route of entry for lead (Pb) into the body is through the gastrointestinal track (96). The latter for example occurs when Pb contaminated clay (kaolin) is consumed. An author studied the implications of consuming such clay in albino rats revealing high concentrations of this metal in the liver and the blood as evidence of its intoxication (92).

In humans, absorption of Pb after ingestion is through the same pathway (gastrointestinal). Its absorption varies with the current physiology of the exposure subject, such as Fe status, available Ca, fasting or age. Lead (Pb) absorption also varies with properties such as solubility, mineralogy particle size etc of the lead ingested (125). A rat study using to Pb acetate (100%) as reference, showed the following absorption: 164% for Pb carbonate; 121% for Pb thallate; 62-67% for Pb sulfide, Pb naphthenate, and Pb octoate; 44% Pb chromate and 14% metallic Pb (126).

Lead (Pb) is known to bind to plasma or red blood cells proteins when it reaches the bloodstream. Only a bit of it is transported in the form of free ions. It is then from blood tissue that it is carried to other tissues and it concentrations in these tissues depend on their vascularization and metabolism (127). Human beings do not have any mechanisms specific to Pb management. Lead (Pb) is distributed into human system, because its ionic characteristics more often than not coincide with metal ions that are essential to man's system. The common pole of metals that are distributed into the human system in a similar manner like Pb include cadmium (Cd), mercury(Hg) and aluminum (Al). The toxicity of these metals is based on a partial ionic-molecular mimicry to other ions essential to human. The toxic mechanism of Pb has been confirmed by authors, who reported that Pb^2+^ has the ability to substitute, at the molecular level for other polyvalent cations like Ca^2+^ and Zn^2+^ (128). The transport mechanisms for these metals necessary for human system is used by Pb for penetration into the system (129, 130). It has been observed that the Ca^2+^ binding sites used by Pb have specific characteristics. For instance, they are wide and evenly charged (131, 132). Many of the zinc-binding sites that are occupied by Pb are formed by sulfur or nitrogen atoms with low coordination numbers (131).

Garza et al. explained that these mimicry interactions of Pb make it interfere with many significant biological processes such as protein maturation, metal transport, ionic conduction, intracellular signaling etc (128). These authors also observed that signaling molecular structures and transport through the membrane are the most affected with Pb neurotoxicity being the outcome. The brain or central nervous system undergo alterations. This is why a prolonged exposure to Pb can end up affecting behavior and walking abilities. The mechanisms by which this happens are complex, but are said to include alterations in the synthesis of neurotransmitters with an eventual damage to neural cells such as astroglia and oligodendrocyte (133). The part of the brain that is responsible for memory and learning in human is mostly the one that manifest greater damage in prolonged Pb exposure (125).

This Pb ability to penetrate human system by substituting for Ca, Zn, and other cations having its ionic form causes Pb to affect the entire environment of the cell, damaging it. It has been proven that these effects result from complex processes engendered by Pb presence in the cell such as genetic regulation, protein synthesis etc or by Pb direct interaction with cell constituents. This scenario, for example, has been observed with some cellular matrix proteoglycans (134, 135) or cell adhesion mediated signals structures that are very important for cell survival but are affected by Pb during their synthesis or maturation (136, 137). Heme-group synthesis has also been observed to be adversely affected by prolonged Pb exposure because of the inhibition of aminolevulinic acid dehydratase (ALAD) a Zn containing enzyme (133). A similar situation occurs when Pb promotes the permeability of the mitochondrial causing the release into the cytoplasm of redox-active proteins such as cytochrome C with cell death as consequence (138).

Lead (Pb), Cd, Hg and Zn are specifically attracted by thiol groups in proteins (139, 140). Prolonged Pb exposure has shown that there is greater affinity for Pb to some proteins molecules more than some essential cations. For instance, ALAD has a greater affinity for Pb than for Zn (usually 2–4) (141–143).

## Conclusion

Considering the outcome of this review, there is need for clay or soil characterization and beneficiation for healthy geophagic practices across ecological context and taxa in order to pronounce on their potential toxicity or benefits. Knowledge of the geochemistry of clays might not be sufficient to aid infer the likely adverse health effects as there is need for bioavailability studies of the elements in the various clay types in various contexts to assess the effect of ingestion of the geophagic materials on the geophagic individuals. Conflicting viewpoints regarding the beneficial and consequences of geophagy are still persisting.

## Recommendations

Clinical control trials are globally persisting as a gap. We strongly recommend clinical control trials on the potential toxicity of kaolin in order to address the conflicting view points and facilitate good policies.

## Author contributions

All authors listed have made a substantial, direct, and intellectual contribution to the work and approved it for publication.

## Conflict of interest

The authors declare that the research was conducted in the absence of any commercial or financial relationships that could be construed as a potential conflict of interest.

## Publisher's Note

All claims expressed in this article are solely those of the authors and do not necessarily represent those of their affiliated organizations, or those of the publisher, the editors and the reviewers. Any product that may be evaluated in this article, or claim that may be made by its manufacturer, is not guaranteed or endorsed by the publisher.
