# Supplementary figures and images for: Geophagia: Benefits and potential toxicity to human—A review
Source: Front Public Health. 2022 Jul 26;10:893831. doi: 10.3389/fpubh.2022.893831 (PMC9360771; doi:10.3389/fpubh.2022.893831)

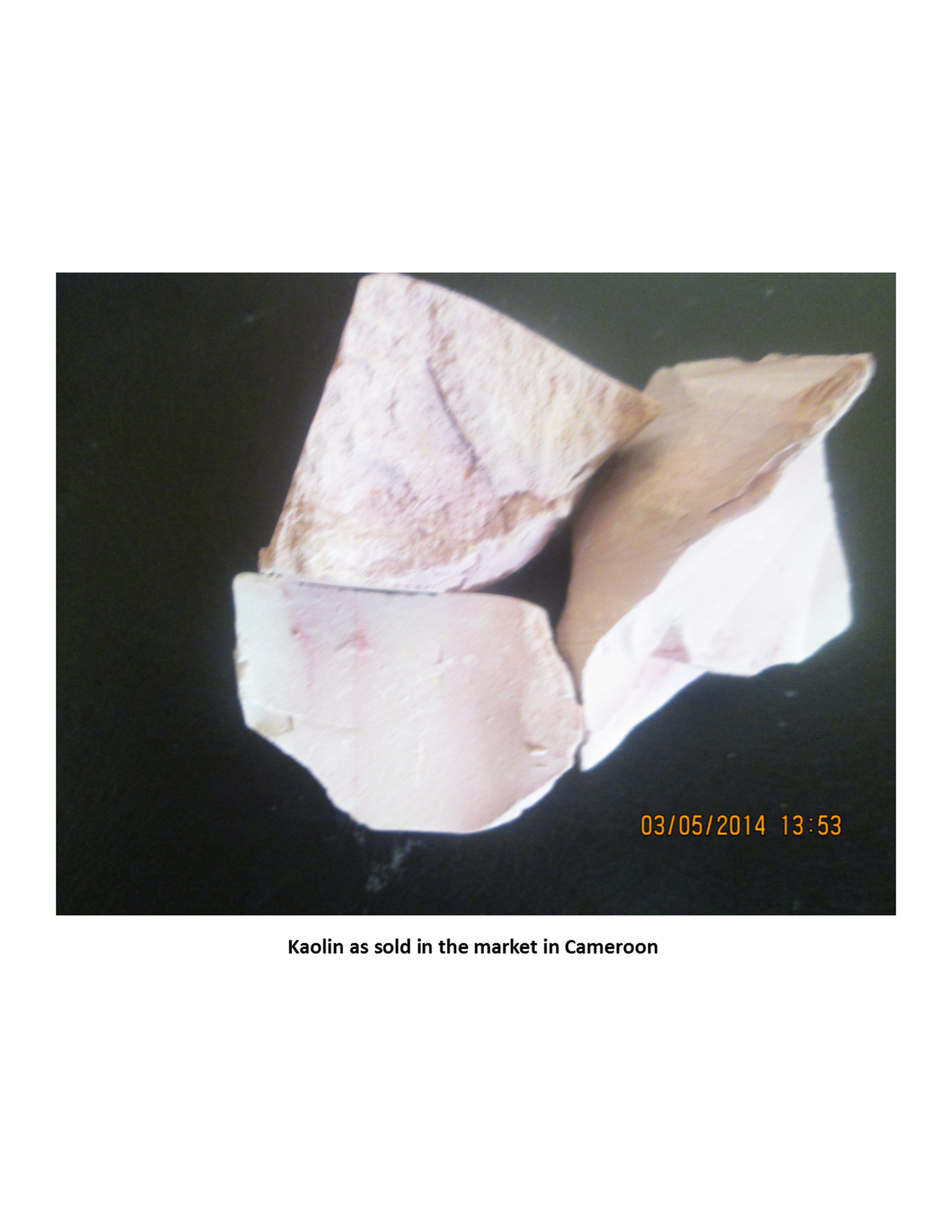

Supplement: Supplementary file 1 [file Image_1.TIF]

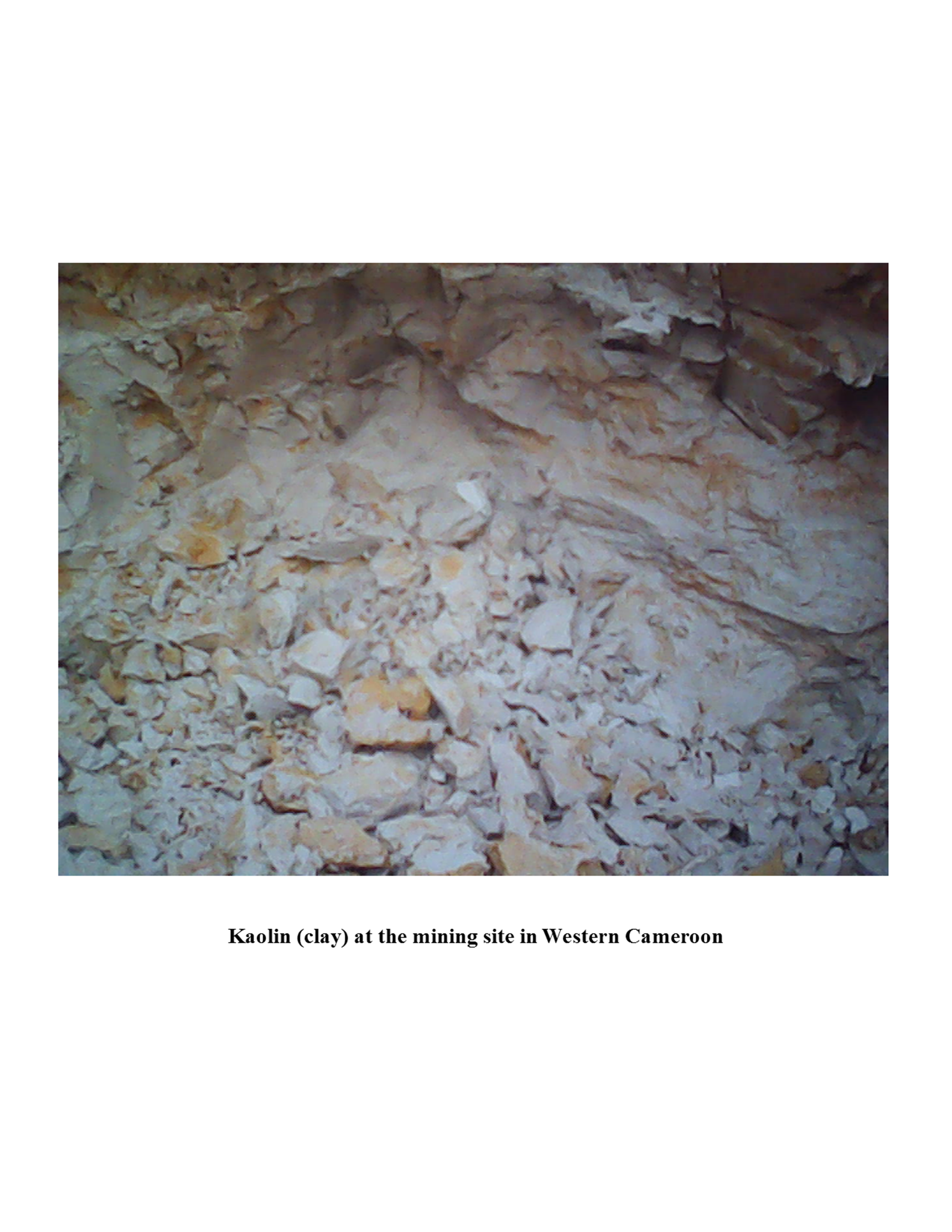

Supplement: Supplementary file 2 [file Image_2.TIF]
